# Supplementary material for: Mechanoresponsive Material of AIE-Active 1,4-Dihydropyrrolo[3,2-b]pyrrole Luminophores Bearing Tetraphenylethylene Group with Rewritable Data Storage
Source: Molecules. 2018 Dec 10;23(12):3255. doi: 10.3390/molecules23123255 (PMC6321101; doi:10.3390/molecules23123255)
Supplement: Supplementary file 1 [file molecules-23-03255-s001.pdf]

Mechanoresponsive material of AIE-active 1,4-dihydropyrrolo[3,2-*b*]pyrrole luminophores bearing tetraphenylethylene group with rewritable data storage

Yuqing Ma, Yuyang Zhang, Lin Kong, Jiaxiang Yang*

[a]*College of Chemistry & Chemical Engineering, Anhui University, Anhui Province Key Laboratory of chemistry for Inorganic/Organic Hybrid Functional Materials, Hefei 230601, P. R. China.*

**Corresponding authors. Tel: +86-0551-63861279; E-mail address: jxyang@ahu.edu.cn*

SUPPLEMENTARY DATA

Contents

1. Figure S1. ^{1}H NMR spectrum of **APPCN**

2. Figure S2. MALDI-TOF spectrum of **APPCN**

3. Figure S3. ^{1}H NMR spectrum of **APPTPECN**

4. Figure S4. ^{13}C NMR spectrum of **APPTPECN**

5. Figure S5. MALDI-TOF spectrum of **APPTPECN**

6. Figure S6. DLS plot of **APPTPECN**

7. Figure S7. Fluorescence decay curves for solid-state of **APPTPECN** before and after grinding

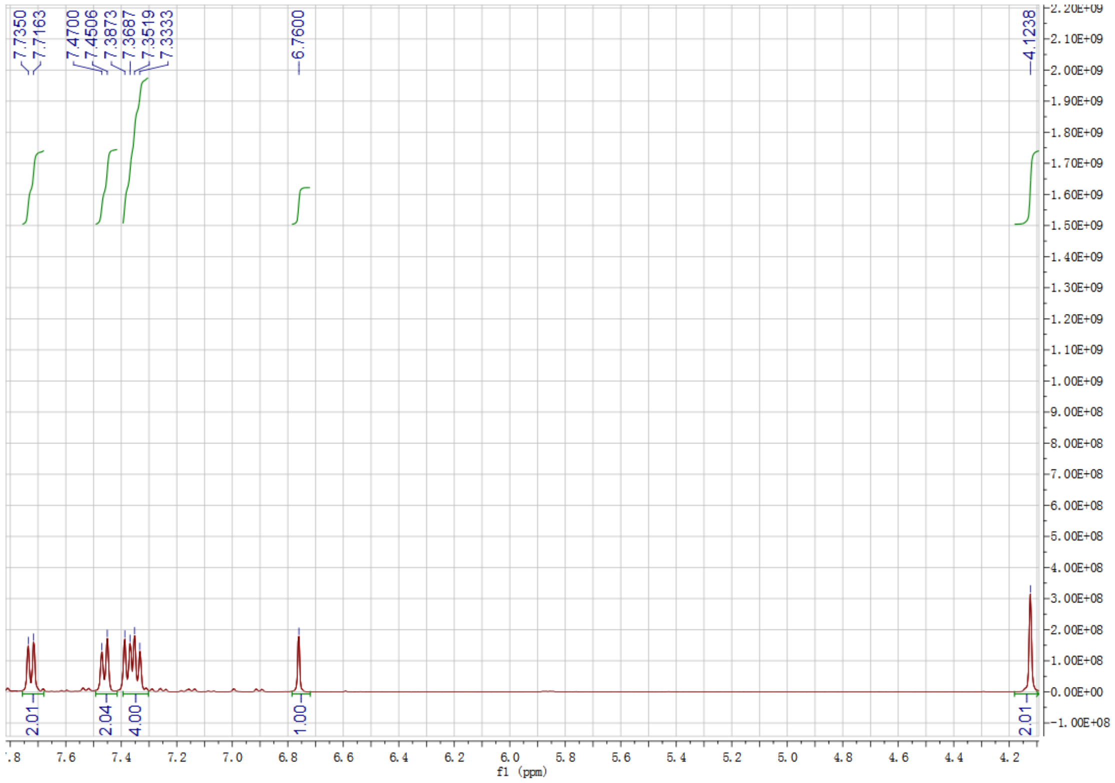

Figure S1. ^{1}H NMR spectrum of **APPCN**

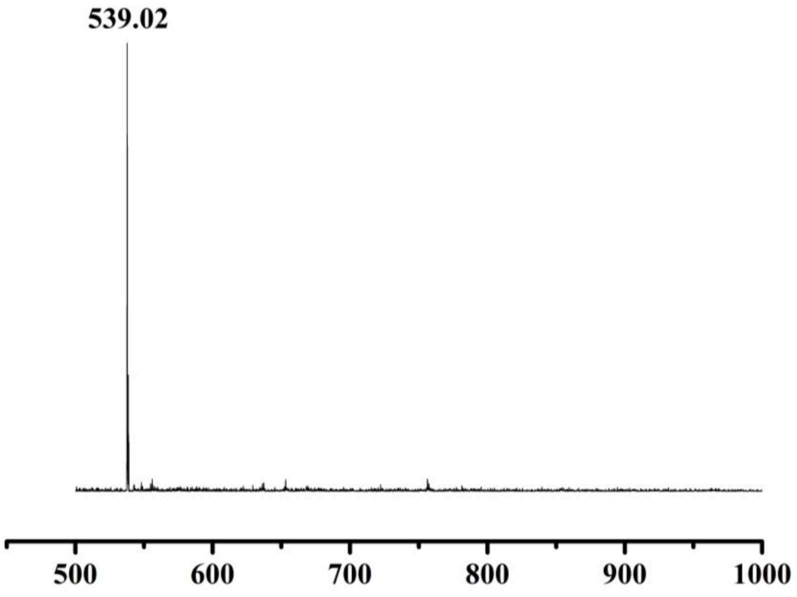

Figure S2. MALDI-TOF spectrum of **APPCN**

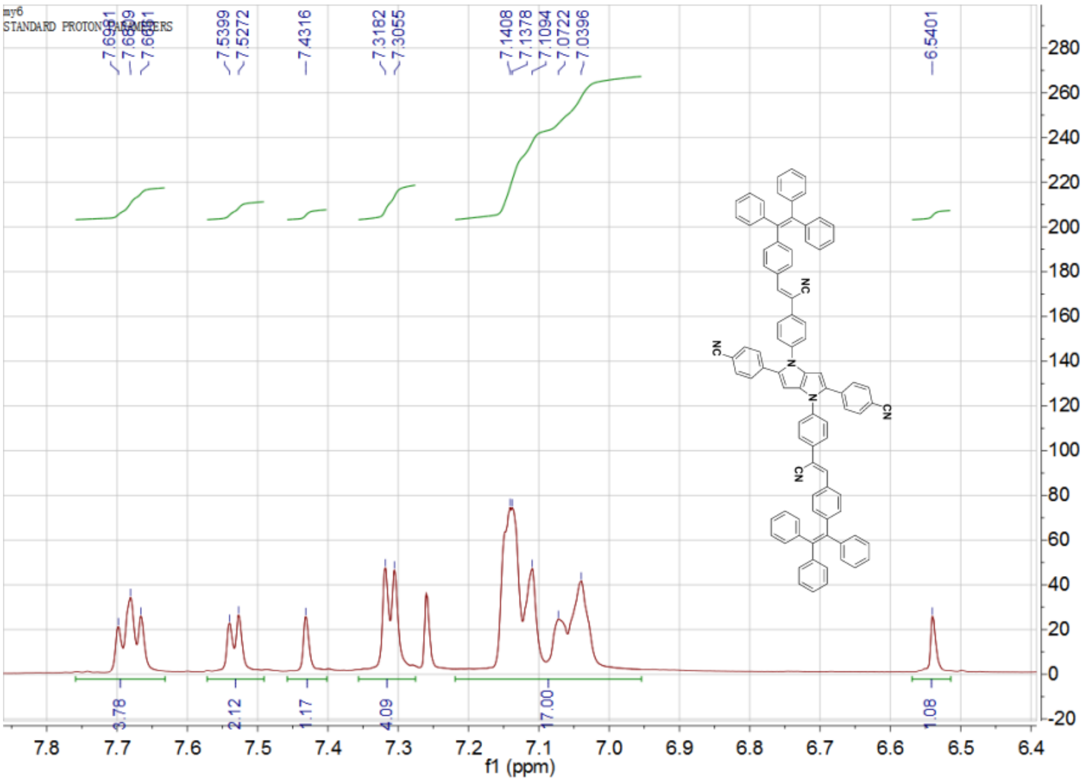

Figure S3. ^{1}H NMR spectrum of **APPTPECN**

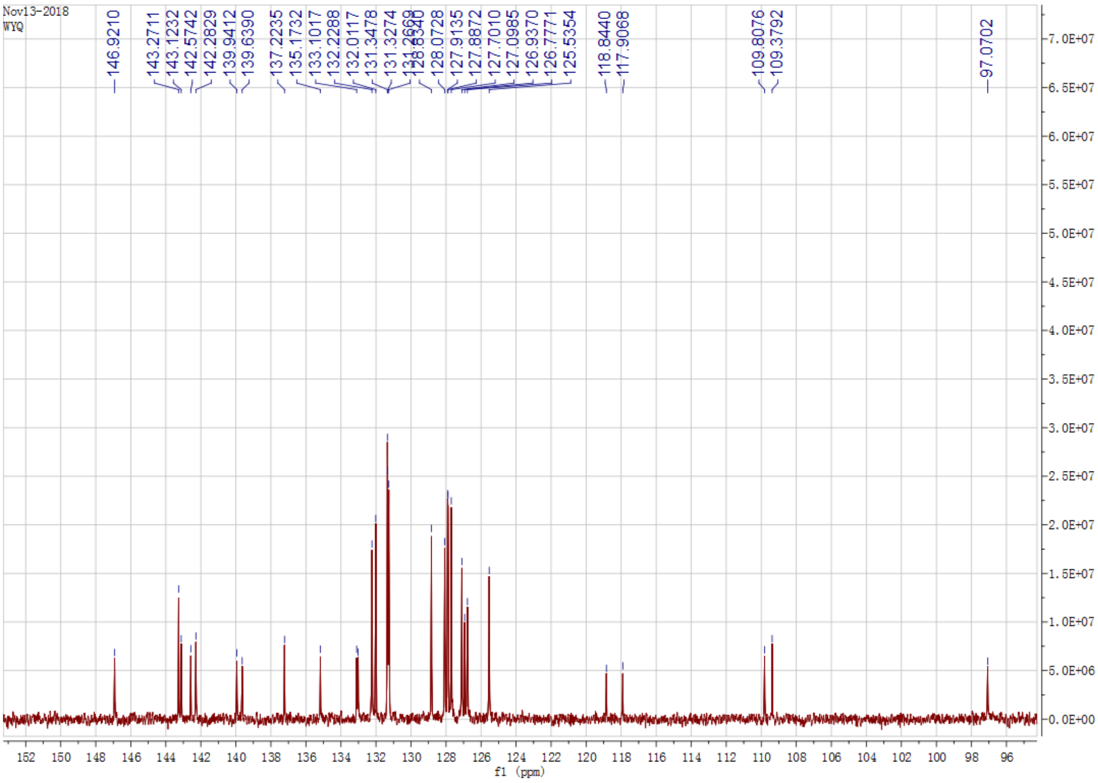

Figure S4. ^{13}C NMR spectrum of **APPTPECN**

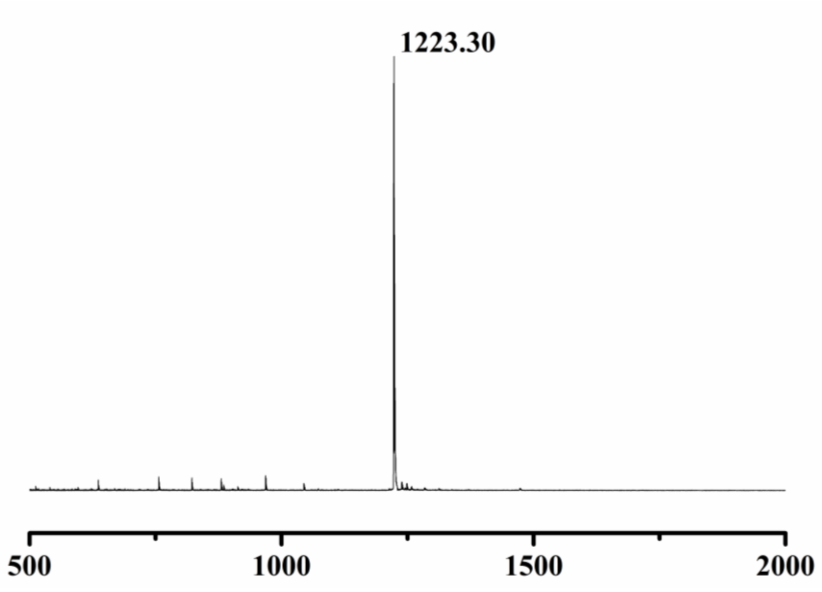

Figure S5. MALDI-TOF spectrum of **APPTPECN**

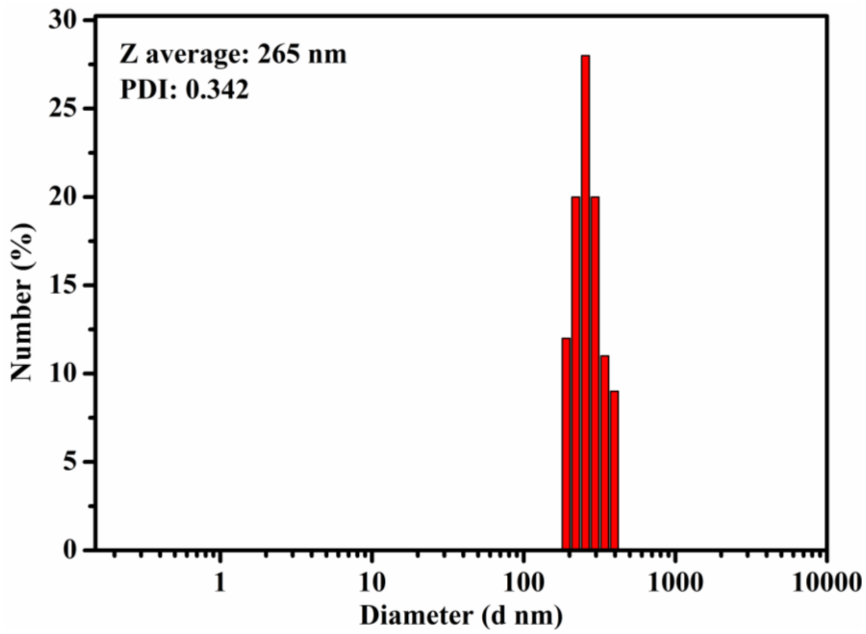

Figure S6. DLS plot of **APPTPECN**

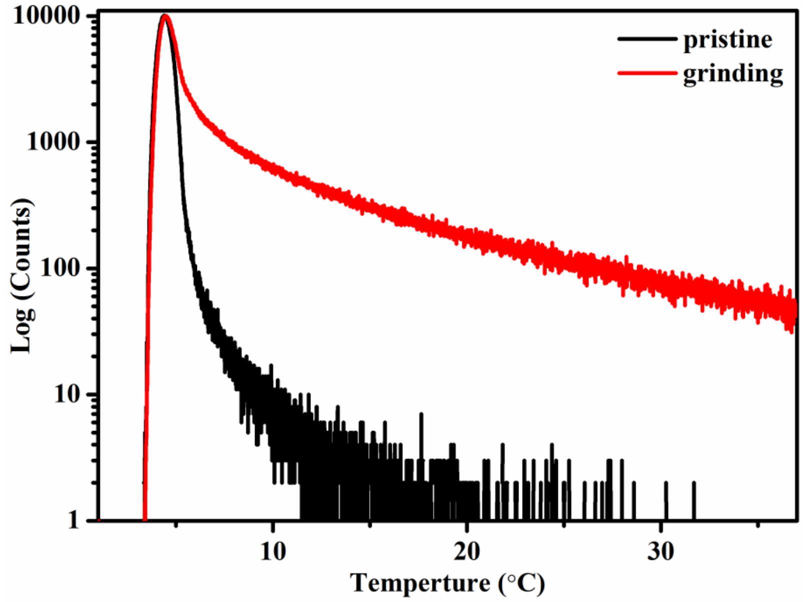

Figure S7. Fluorescence decay curves for solid-state of **APPTPECN** before and after grinding
